# Protective effects of methanolic extract of *Juglans regia L.* leaf on streptozotocin-induced diabetic peripheral neuropathy in rats

**DOI:** 10.1186/s12906-017-1983-x

**Published:** 2017-10-02

**Authors:** Davood Nasiry, Ali Reza khalatbary, Hassan Ahmadvand, Fereshteh Talebpour Amiri, Esmaeil Akbari

**Affiliations:** 1 Molecular and Cell Biology Research Center, Department of Anatomy, Faculty of Medicine, Mazandaran University of Medical Sciences, Sari, Iran; 2 Department of Biochemistry, Faculty of Medicine, Lorestan University of Medical Sciences, Khorramabad, Iran; 3 Razi Herbal Researches Center, Lorestan University of Medical Sciences, Khorramabad, Iran; 4 Immunogenetic Research Center and Department of Physiology and Pharmacology, Faculty of Medicine, Mazandaran University of Medical Sciences, Sari, Iran

**Keywords:** Diabetic neuropathy, *Juglans regia L.* leaf, Hyperglycemia, Antioxidant, Rats, Streptozotocin

## Abstract

**Background:**

Oxidative stress has a pivotal role in the pathogenesis and development of diabetic peripheral neuropathy (DPN), the most common and debilitating complications of diabetes mellitus. There is accumulating evidence that *Juglans regia L.* (*GRL*) leaf extract, a rich source of phenolic components, has hypoglycemic and antioxidative properties. This study aimed to determine the protective effects of *Juglans regia L.* leaf extract against streptozotocin-induced diabetic neuropathy in rat.

**Methods:**

The DPN rat model was generated by intraperitoneal injection of a single 55 mg/kg dose of streptozotocin (STZ). A subset of the STZ-induced diabetic rats intragastically administered with *GRL* leaf extract (200 mg/kg/day) before or after the onset of neuropathy, whereas other diabetic rats received only isotonic saline as the same volume of *GRL* leaf extract. To evaluate the effects of *GRL* leaf extract on the diabetic neuropathy various parameters, including histopathology and immunohistochemistry of apoptotic and inflammatory factors were assessed along with nociceptive and biochemical assessments.

**Results:**

Degeneration of the sciatic nerves which was detected in the STZ-diabetic rats attenuated after *GRL* leaf extract administration. Greater caspase-3, COX-2, and iNOS expression could be detected in the STZ-diabetic rats, which were significantly attenuated after *GRL* leaf extract administration. Also, attenuation of lipid peroxidation and nociceptive response along with improved antioxidant status in the sciatic nerve of diabetic rats were detected after *GRL* leaf extract administration. In other word, *GRL* leaf extract ameliorated the behavioral and structural indices of diabetic neuropathy even after the onset of neuropathy, in addition to blood sugar reduction.

**Conclusion:**

Our results suggest that *GRL* leaf extract exert preventive and curative effects against STZ-induced diabetic neuropathy in rats which might be due to its antioxidant, anti-inflammatory, and antiapoptotic properties.

**Graphical abstract:**

Protection against neuropathy
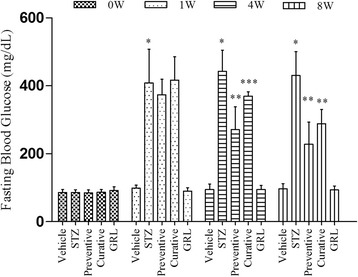

## Background

Peripheral neuropathy is one of the most common long term complications of diabetes mellitus which affects all peripheral nerves, including pain fibers [1]. Almost 12% of all diabetic patients are affected with symptomatic painful diabetic neuropathy [1]. In this regard, several mechanisms account for the development of diabetic neuropathy such as metabolic disorders, microvascular damages, neurotrophic support deficit, alternation in neuro-immune interactions, neural and glial cell apoptosis, and inflammation [2, 3]. It is well known that most of these factors result from increased oxidative stress which has a pivotal role not only in the development of diabetic neuropathy but also in the resistance of it even after good glycemic control, might be due to accumulated free radicals that are not easily removed [4]. Increased oxidative stress after hyperglycemia is caused mainly due to autooxidative glycosylation, advanced glycation end-product (AGE) formation, and increased polyol pathway activity [2]. Therefore, it has been postulated that the use of antioxidant supplements may offer some protection against this complication through scavenging of the free radicals. In recent decades, a rapidly growing number of natural phenolic compounds with free radical scavenging properties have been described. The seeds, green husks, and leaves of the Persian or common walnut (*Juglans regia L.*), the best-known member of the *Juglans* genus, are a rich source of these molecules which have been traditionally used in Iranian folk medicine for treatment of several diseases such as infections, inflammations, and diabetes and its complications [5, 6]. Interestingly, these effects were confirmed according to the recent scientific studies [7, 8]. Flavonoids, phenolic acids, and naphtoquinones are considered as major phenolic compounds in *Juglans regia L.* leaves [9, 10]. Pharmacologically, there is accumulating evidence that attributed the beneficial effects of *Juglans regia L.* leaf extract to a variety of biological activities, including anti-oxidative [8, 10], anti-inflammatory [11], anti-carcinogenic [12], anti-microbial [13], and anti-fungal properties [14]. Recently, there are a few experimental studies on the hypoglycemic effect of *Juglans regia L.* leaf extract in diabetes mellitus (15–18). These studies documented that administration of *Juglans regia L.* leaf extract significantly reduced fast blood sugar (FBS) and hemoglobin A1c (HbA1c) compared to control groups [15–18]. Moreover, results of two clinical trial studies have shown that fast blood glucose (FBG) and HbA1c significantly decreased after consumption of 100 mg *Juglans regia L.* leaf extract for 3 months [19] and 200 mg *Juglans regia L.* leaf extract for 2 months [7] compared to placebo groups. An in vitro study also reported that walnut leaf extract inhibits protein tyrosine phosphatase 1B (PTP1B) and enhances glucose-uptake [20]. In this study, we investigated the beneficial effects and the underlying molecular mechanisms of *Juglans regia L.* leaf extract in diabetic neuropathy as a common serious complication of diabetes, according to the traditional claims and the existing scientific evidences regarding hypoglycemic and antioxidative properties of *Juglans regia L.* leaf extract.

## Methods

Extract preparation and gas chromatography-mass spectrometry (GC-MS).

Fresh leaves of *Juglans regia L.* were collected during July–August 2014 from cultivated trees in Khorramabad (Lorestan province, western Iran) and authenticated by Natural Resources Research Center of Lorestan Province. It was registered under the voucher number 8763. Briefly, the leaves were dried and pulverized, and then stored in dark at room temperature. Methanol was added to the pulverized leaves for 72 h and then filtered through filter paper. The obtained extracts were concentrated under reduced pressure at 40 °C. GC-MS was carried out using a Hewlett-Packard 6859 with a quadrupole detector, on a HP-5 column, operating at 70 eV ionization energy, using the same temperature programme and carrier gas as above. Retention indices were calculated by using retention times of n-alkanes that were injected after the extract at the same chromatographic to Van Den Dool method [21].

### Animals

Male adult Spargue-Dawley rats were used (250–275 g) (laboratory animal research center, Sari, Iran) in this study. They were kept in the laboratory under constant conditions of temperature (23 ± 1 °C) and light/dark cycle (12 h/12 h) for at least 7 days before and over the course of the experiment. All experimental procedures and protocols were approved by ethical committee of Health Sciences, Mazandaran University of Medical Sciences (Ethics approval references number: IR.MAZUMS.REC.95.2266) to minimize the animal’s suffering. These guidelines were in accordance with the internationally accepted principles for the care and use of laboratory animals.

### Induction of diabetes and experimental design

Diabetes was induced by an intraperitoneal injection of 55-mg/kg [22] single dose of streptozotocin (Santa Cruz Biotechnology) diluted in 0.1 M citrate buffer with PH-4.5. Blood samples were collected from tail vein ~48 h after streptozotocin administration and estimated plasma glucose levels using a commercial glucometer and test strips (Accu-Chek® Active test meter). Rats with plasma glucose level more than 250 mg/dLwere considered as diabetics and were further considered for study. *Juglans regia L.* leaf extract was administered by oral gavages (200-mg/kg/day). The doses and treatment schedules were based on previous studies [15, 17, 22–24] and pilot experiments in our laboratory.

The animals were randomly allocated in five groups, each containing 7 rats: (Ι) Streptozotocin (STZ) treated group, which received an intraperitoneal injection of 55-mg/kg single dose of streptozotocin diluted in 0.1 M citrate buffer and then received isotonic saline as the same volume of *GRL* leaf extract; (ΙΙ) Preventive group, which received STZ and then received orally administration of 200-mg/kg/day *Juglans regia L.* leaf extract from the 1st week and continued up to the end of the 8th week after induction of diabetes; (III) Curative group, which received STZ and then received orally administration of 200-mg/kg/day *Juglans regia L.* leaf extract from the 5th week and terminated at the end of the 8th week after induction of diabetes; (IV) Vehicle group, which received only buffer citrate as the same volume of STZ (non-diabetic rats); (V) *Juglans regia L.* leaf extract treated group (GRL group, non-diabetic rats) which received only 200-mg/kg/day *Juglans regia L.* leaf extract. All animals in the experimental groups survived until the end of the experiments, except one rat of the Streptozotocin treated group (Ι). Blood glucose level and body weight were measured on weeks 0, 1, 4, and 8 after STZ injection.

### Formalin test

The formalin test was carried out in all groups on weeks 0, 1, 4, and 8 after STZ or vehicle injection. The rats were given a subcutaneous injection of 2.5% formalin into the plantar surface of one hind paw and scoring of nociceptive behaviours began immediately after formalin injection (each 3 min time block) and continued for 60 min. Average nociceptive score, ranging from 0 (the injected paw is not favored) to 3 (the injected paw is licked, bitten or shaken), was measured [25]. At the end of the eighth week, the rats immediately after the last formalin test were euthanized with an injection of sodium pentobarbital and then sciatic nerves were harvested for histopathological, immunohistochemical, and biochemical assessments.

### Biochemistry

The obtained samples (the left side sciatic nerves) were thoroughly cleaned of blood, then were immediately frozen and stored in a − 80 °C freezer for assays of tissue malondialdehyde (MDA) and Glutathione (GSH) levels, catalase (CAT) and superoxide dismutase (SOD) activities. The estimation of MDA level was done by measuring thiobarbituric acid reactive substances (TBARS) which was determined spectrophotometrically by the absorbance at 535 nm [26]. The concentration was expressed as micromoles per milligram of protein. The estimation of GSH level was done with 10% trichloroacetic acid as described by Akerboom and Sies [27] which was determined spectrophotometrically by the absorbance at 412 nm. The concentration was expressed as micromoles per milligram of protein. The estimation of CAT activity was determined spectrophotometrically by following the decrease in absorption at 240 nm in a reaction medium containing phosphate buffer and hydrogen peroxide [28] and expressed as nanomoles trolox equivalent per milligram of protein. The estimation of SOD activity was based on the inhibition of superoxide radical reaction with pyrogallol [29] which was determined spectrophotometrically by the absorbance at 420 nm and expressed as nanomoles trolox equivalent per milligram of protein.

### Histopathology

The obtained samples (the right side sciatic nerves) were immediately fixed in 10% formaldehyde and embedded in paraffin. Three-micrometer serial sections were taken from the paraffin-embedded blocks by microtome. Some tissue sections were deparaffinized and stained with hematoxylin and eosin (H&E) or luxol fast blue (LFB), and studied by light microscopy to assess the histopathological changes. Morphometric analysis of the sciatic nerve was done using ImageJ software (MacBiophotonics ImageJ 1.41a) [30]. All the histological assessments were done in a blinded fashion.

### Immunohistochemistry

For immunohistochemistry, some sections were incubated in Goat normal serum (in order to block non-specific site), and then with anti-caspase 3 rabbit polyclonal antibody (1:100 in PBS, *v*/v, ABCAM ab4051), anti-COX 2 rabbit polyclonal antibody (1:100 in PBS, v/v, ABCAM ab15191), anti-S100B rabbit polyclonal antibody (1:1000 in PBS, v/v, ABCAM ab868), and anti-iNOS rabbit polyclonal antibody (1:100 in PBS, v/v, ABCAM ab15323) overnight at 4 °C. Sections were washed with PBS and then incubated with secondary antibody conjugated with horseradish peroxidase (goat anti-rabbit IgG, ABCAM ab205718) for 2 h and detected by diaminobenzidine tetrahydrochloride for 5 min. After wards, they were dehydrated and mounted. For negative controls, primary antibodies were omitted. For quantitative analysis, immunohistochemical photographs (n = five photos from each samples collected from all rats in each experimental group) were assessed by densitometry using Image J software. Data are expressed as a percentage of total tissue area.

### Statistical analysis

Statistical analysis was carried out in SPSS (Version 15, Chicago, IL, USA). Results were presented as mean values (±SD). The K-S test was used in order to evaluate the normality of the data. One-way ANOVA followed by Tukey′s post-hoc tests were used to compare each two groups and data among the groups, respectively. Two-way ANOVA followed by Bonferroni’s post-hoc tests were used for multiple comparisons. A value of *p* < 0.05 was considered significant.

## Results

### GC-MS analysis

The various chemical constituents identified in *Juglans regia L.* leaf extract are shown in Table 1. A total of 28 constituents were identified in the leaf extract. The principal components of the extract were 2-ß-Pinene (17.09%), α-Pinene (13.29%), trans-Caryophyllene (10.58%), and Germacrene D (8.90%). Other minor identified constituents were dl-Limonene (3.85%), Terpine-4-ol (3.70%), ß-Slinene (3.25%), and Methyl salicylate (3.07%).

**Table 1:** Chemical composition of the Juglans regia L. (GRL) leaf extract

Retention time (min)	Compound name	Area	%
4.99	alpha-Pinene	6,609,366	13.29
5.3	Camphene	273,403	0.55
5.89	2-beta-Pinene	8,499,378	17.09
6.12	beta-Myrcene	750,812	1.51
6.96	alpha-Terpinene	428,650	0.86
7.04	dl-Limonene	1,912,844	3.85
7.1	1,8-Cineole	1,237,434	2.49
7.77	gamma-Terpinene	539,310	1.08
10.29	Terpine-4-ol	1,841,560	3.70
10.87	Methyl salicylate	1,527,680	3.07
13.96	Bornyl acetate	1,100,607	2.21
15.41	alpha-Copaene	857,830	1.72
16.37	Aromandenrene	556,457	1.12
16.59	Tetradecane	1,302,170	2.62
17.27	trans-Caryophyllene	5,263,715	10.58
17.57	Alpha-Humulene	1,285,523	2.58
18.08	Germacrene D	4,428,268	8.90
18.67	beta-Cubebene	520,099	1.05
18.81	beta-Selinene	1,615,286	3.25
19.08	Pentadecane	1,419,619	2.85
19.18	Bicyclogermacrene	978,555	1.97
19.62	alpha-Amophene	544,869	1.10
19.8	delta-Cadinene	1,255,420	2.52
20.2	Dihydroactinidiolide	954,467	1.92
21.27	Spathulenol	1,393,102	2.80
21.33	Caryophyllene oxide	860,467	1.73
21.44	Hexadecane	685,834	1.38
23.81	Nonacosanol	1,098,357	2.21

### Blood glucose levels and body weight

**Fig. 1:**
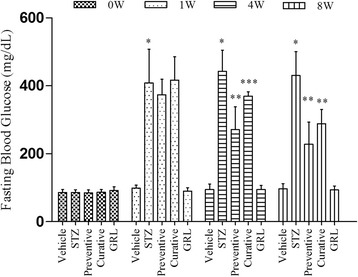
Effects of *GRL* on fasting blood glucose (FBS). Histogram shows the levels of FBS in all groups. Values are expressed as milligrams per deciliter (mg/dL) of 7 rats/group. *P < 0.001 versus vehicle group and *GRL* group; **P < 0.001 versus STZ group; ***P < 0.05 versus Preventive group by one-way ANOVA followed by Tukey′s post-hoc tests and two-way ANOVA followed by Bonferroni’s post-hoc tests

The histogram of the fasting blood sugar (FBS) levels for all groups is shown in Fig. 1. Administration of STZ in the STZ group produced a significant elevation (*p* < 0.001) in FBS level and the hyperglycemia was maintained throughout experimental period compared to vehicle group. At the end of the experiment, the FBS levels in the Preventive and Curative group were significantly lower than that those in the STZ group (p < 0.001), while the differences between Preventive and Curative were not significant (*p* > 0.05).

Body weight of 8th week diabetic rats was significantly (*p* < 0.001) lower than vehicle rats (Table 2), while the differences between STZ and Preventive or Curative treatment group were not significant (*p* > 0.05).

**Table 2:** Effect of the Juglans regia L. (GRL) leaf extract on body weight

Experimental Groups	Body weight (g)	Body weight (g)	Body weight (g)	Body weight (g)
	0 week	1 week	4 week	8 week
Vehicle	269.70 ± 6.65	288.30 ± 27.79	326.30 ± 14.84	337.70 ± 6.80
STZ	260.70 ± 8.50	253.70 ± 6.65	246.30 ± 11.15^*^	224.00 ± 24.27^*^
Preventive	266.00 ± 11.27	276.70 ± 7.23	233.70 ± 10.50	229.50 ± 6.36
Curative	274.00 ± 1.37	264.00 ± 16.52	250.70 ± 22.50	224.70 ± 19.09
GRL	267.00 ± 9.89	295.00 ± 15.87	304.70 ± 13.61	316.00 ± 7.81

Data are represented in Mean ± SD of 7 rats/group

^*^ *p* < 0.001 versus vehicle and GRL groups by one-way ANOVA followed by Tukey′s post-hoc tests. Time (week) after STZ induction

### Nociceptive response

**Fig. 2:**
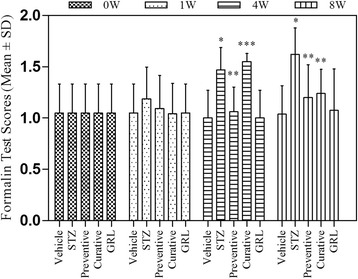
Effects of *GRL* on nociceptive response. Histogram shows the levels of nociceptive response in all groups. Values are mean ± SD of 7 rats/group. *P < 0.001 versus vehicle and *GRL* group; **P < 0.001 versus STZ group; ***P < 0.001 versus Preventive group by one-way ANOVA followed by Tukey′s post-hoc tests and two-way ANOVA followed by Bonferroni’s post-hoc tests

The nociceptive rating scores in the five groups have been presented as mean value ± SD (Fig. 2). The nociceptive response between the all groups before and one week after induction of diabetes were not significant. The nociceptive response of the STZ-treated diabetic rats at the end of the fourth week was found to be significantly (*p* < 0.001) increased as compared to vehicle rats, while treatment with the leaf extract in Preventive group significantly (*p* < 0.001) decreased the nociceptive score compared to the STZ-treated group. There was a significant difference (*p* < 0.001) in the nociceptive score at the end of the study (8th week) between STZ and Preventive or Curative treatment group, while the differences between Preventive and Curative were not significant (*P* < 0.05).

### Biochemical analysis

Malondialdehyde (MDA) levels for all groups at the end of the experiment are shown in Table 3. Administration of STZ in the STZ group produced a significant elevation (*p* < 0.001) in lipid peroxidation level compared to vehicle group. The MDA levels in the Preventive and Curative group were significantly lower than that those in the STZ group (*p* < 0.01), while the differences between Preventive and Curative group were not significant (*p* > 0.05).

**Table 3:** Effect of the Juglans regia L. (GRL) leaf extract on biochemical markers of rat sciatic nerve affected by STZ-induced diabetic neuropathy

Experimental Groups	MDA	GSH	CAT	SOD
	μmol/mg-protein	μmol/mg-protein	unit/mg-protein	unit/mg-protein
Vehicle	35.08 ± 4.44	23.67 ± 1.15	10.40 ± 0.52	35.67 ± 2.51
STZ	79.72 ± 4.89^*^	13.84 ± 1.03^*^	5.43 ± 1.60^*^	20.27 ± 1.81^*^
Preventive	57.30 ± 6.25^**^	17.95 ± 0.54^**^	9.13 ± 0.10^**^	24.92 ± 5.15
Curative	52.45 ± 7.95^**^	16.94 ± 1.21^***^	9.34 ± 0.56^**^	20.64 ± 2.52
GRL	32.33 ± 5.85	24.00 ± 1.00	10.55 ± 1.24	36.67 ± 2.08

Data are represented in Mean ± SD of 7 rats/group

^*^ *p* < 0.001 versus vehicle and GRL group

^**^ *p* < 0. 01 versus STZ group

^***^ *p* < 0.05 versus STZ group by one-way ANOVA followed by Tukey′s post-hoc tests

Glutathione (GSH) levels for all groups at the end of the experiment are shown in Table 3. Administration of STZ in the STZ group produced a significant (*p* < 0.001) decrease in GSH level compared to vehicle group. The GSH levels in the Preventive and Curative group were significantly (*p* < 0.01) higher than that in the STZ group, while the differences between Preventive and Curative group were not significant (*p* > 0.05).

Catalase (CAT) activity levels for all groups at the end of the experiment are shown in Table 3. Administration of STZ in the STZ group produced a significant (*p* < 0.001) decrease in CAT activity compared to vehicle group. The CAT activities in the Preventive and Curative group were significantly (*p* < 0.01) higher than that in the STZ group, while the differences between Preventive and Curative group were not significant (*p* > 0.05).

Superoxide dismutase (SOD) activity levels for all groups at the end of the experiment are shown in Table 3. Administration of STZ in the STZ group produced a significant (*p* < 0.001) decrease in SOD activity compared to vehicle group. The SOD activities in the Preventive and Curative group were not significantly (*p* > 0.05) higher than that in the STZ group.

### Histopathologic changes

**Fig. 3:**
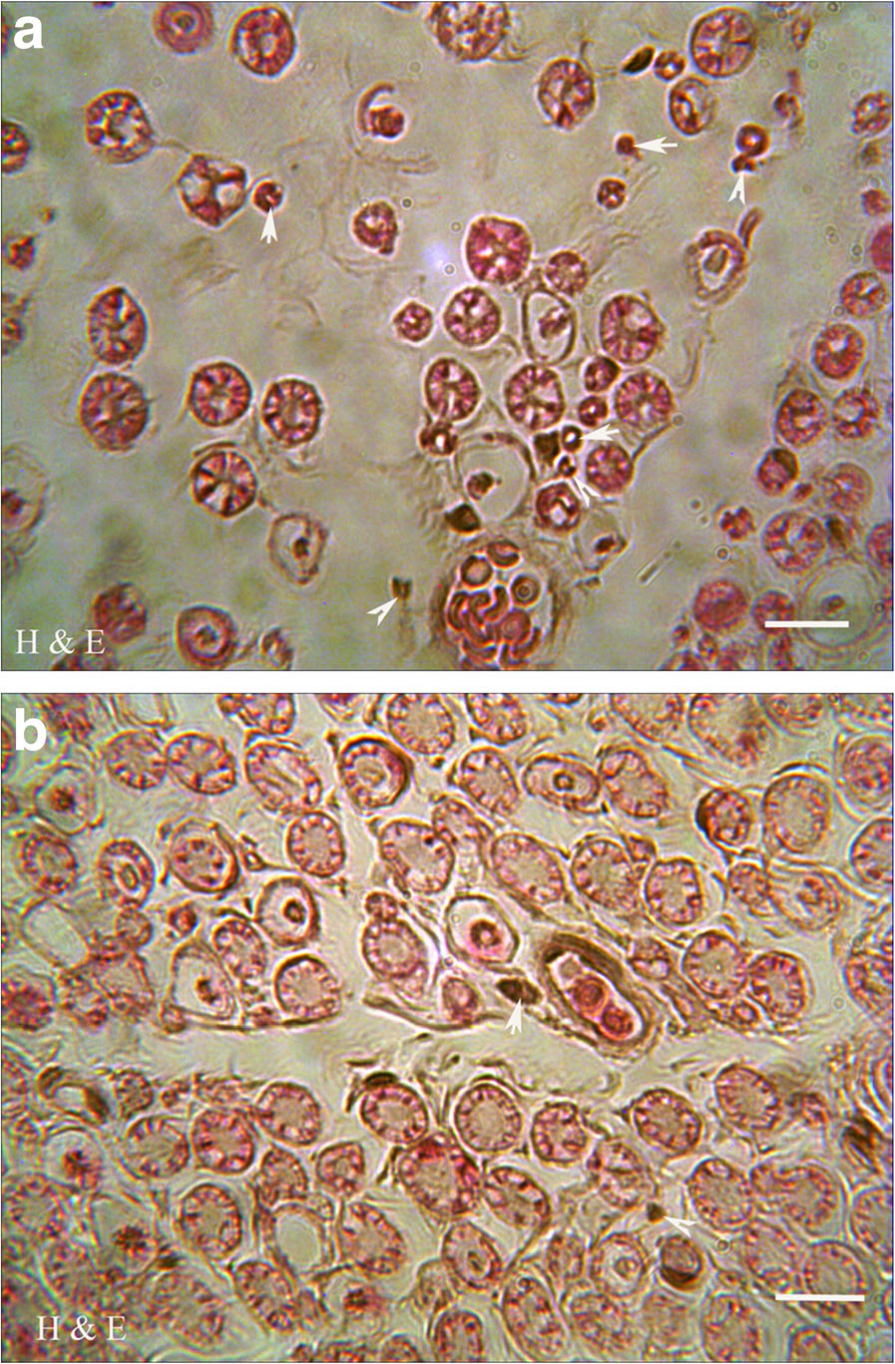
Effects of *GRL* on tissue protection at the end of the study (8th week). Photomicrographs of sciatic horizontal section of STZ group (**a**) with degeneration of the myelin sheath (arrows) and numerous infiltrated inflammatory cells (arrowheads) among the separated nerve fibers, and STZ plus *GRL* groups (**b**) with only focal loss of myelin sheath (arrows) and a few scattered inflammatory cells (arrowheads) (stained with hematoxylin and eosin; original magnification: ×400, bar: 250 μm)

Histological examination of the sciatic nerves in the STZ-induced diabetic animals (Fig. 3a) revealed that the myelin sheath of the myelinated nerve fibers was thin, loose, and disorganized. Meanwhile, numerous inflammatory cells infiltrated among the separated nerve fibers. Treatment with *Juglans regia L.* leaf extract reduced the changes, so that only focal loss of myelin sheath and a few scattered inflammatory cells were detected in Preventive and Curative group (Fig. 3b). No detectable injury was shown in vehicle and *GRL* group. Computer-aided morphometric analyses of the myelinated nerve fibers in the experimental groups are shown in Table 4.

**Table 4:** Morphometric analysis of the sciatic nerve at the end of the 8th week

Experimental Groups	Axon density (N/mm^2^)	Fiber area (μm^2^)	G ratio (axon diameter/fiber diameter)
Vehicle	27.67 ± 8.59	6563 ± 2366	0.37 ± 0.05
STZ	34.40 ± 18.00	4703 ± 1651^*^	0.44 ± 0.07^*^
Preventive	38.53 ± 8.79	5716 ± 1868^***^	0.35 ± 0.06^**^
Curative	32.80 ± 7.62	6012 ± 2323^**^	0.36 ± 0.08^**^
GRL	28.67 ± 8.59	6463 ± 2360	0.38 ± 0.05

Data are represented in Mean ± SD of 7 rats/group

^*^ *p* < 0.001 versus vehicle and GRL group

^**^ *p* < 0.001 versus STZ group

^***^ *p* < 0.05 versus STZ group by one-way ANOVA followed by Tukey′s post-hoc tests

### Immunohistochemical assessment

**Fig. 4:**
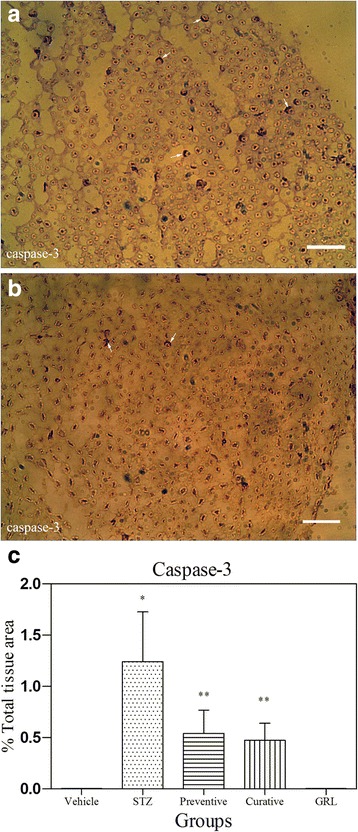
Light Photomicrographs show immunohistochemical expression of caspase-3 in STZ (**a**) and STZ plus *GRL* (**b**) groups (original magnification: ×400, bar: 250 μm). The positive staining of caspase-3 is presented by a brown color of cytoplasm (arrows). Densitometry analysis of immunohistochemical photomicrographs for caspase-3 was assessed. Data are expressed as a percentage of total tissue area (**c**) of 7 rats/group. *P < 0.001 versus vehicle and *GRL* group; **P < 0.001 versus STZ group by one-way ANOVA followed by Tukey′s post-hoc tests

**Fig. 5:**
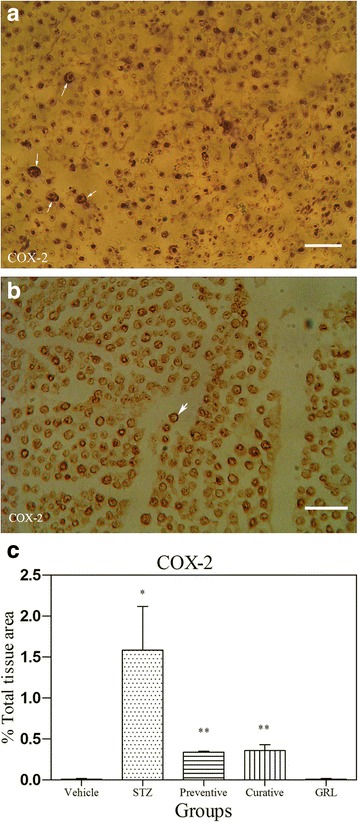
Light Photomicrographs show immunohistochemical expression of COX-2 in STZ (**a**) and STZ plus *GRL* (**b**) groups (original magnification: ×400, bar: 250 μm). The positive staining of COX-2 is presented by a brown color of cytoplasm (arrows). Densitometry analysis of immunohistochemical photomicrographs for COX-2 was assessed. Data are expressed as a percentage of total tissue area (**c**) of 7 rats/group. *P < 0.001 versus vehicle and *GRL* group; **P < 0.001 versus STZ group by one-way ANOVA followed by Tukey′s post-hoc tests

**Fig. 6:**
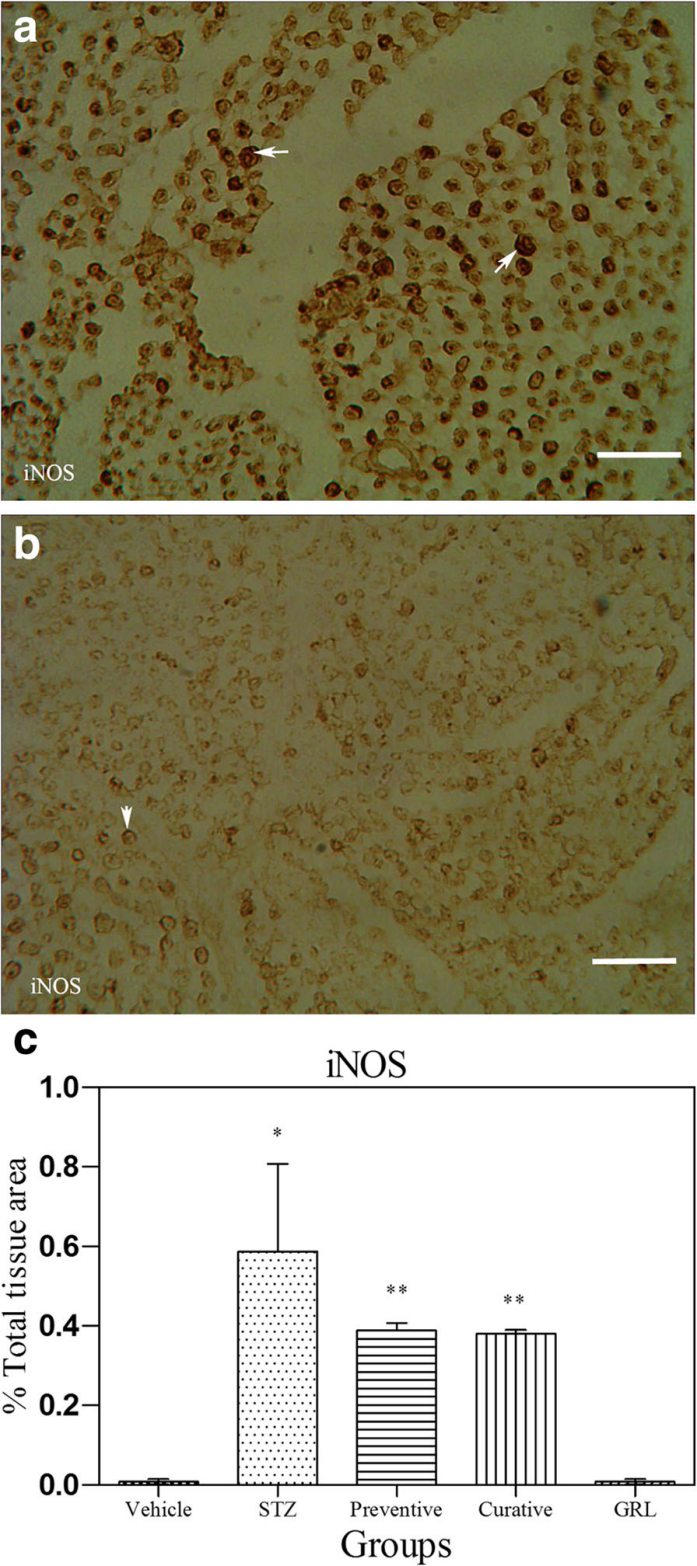
Light Photomicrographs show immunohistochemical expression of iNOS in STZ (**a**) and STZ plus *GRL* (**b**) groups (original magnification: ×400, bar: 250 μm). The positive staining of iNOS is presented by a brown color of cytoplasm (arrows). Densitometry analysis of immunohistochemical photomicrographs for iNOS was assessed. Data are expressed as a percentage of total tissue area (**c**) of 7 rats/group. *P < 0.001 versus vehicle and *GRL* group; **P < 0.05 versus STZ group by one-way ANOVA followed by Tukey′s post-hoc tests

**Fig. 7:**
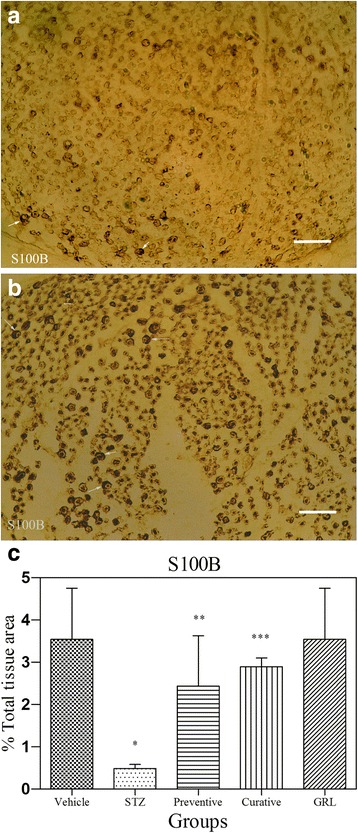
Light Photomicrographs show immunohistochemical expression of S100B in STZ (**a**) and STZ plus *GRL* (**b**) groups (original magnification: ×400, bar: 250 μm). The positive staining of S100B is presented by a brown color of cytoplasm (arrows). Densitometry analysis of immunohistochemical photomicrographs for S100B was assessed. Data are expressed as a percentage of total tissue area (**c**) of 7 rats/group. *P < 0.001 versus vehicle and *GRL* group; **P < 0.05 versus STZ group; ***P < 0.01 versus STZ group by one-way ANOVA followed by Tukey′s post-hoc tests

Figures 4, 5, 6, and 7 show the immunohistochemical staining of caspase-3, COX-2, iNOS, and S100B, respectively. Administration of STZ in the STZ group increased the expression of caspase-3 (Fig. 4a), COX-2 (Fig. 5a), and iNOS (Fig. 6a), while *Juglans regia L.* leaf extract treatment in the Preventive and Curative group reduced the degree of positive staining for caspase-3 (Fig. 4b), COX-2 (Fig. 5b), and iNOS (Fig. 6b) compared to the STZ group. Administration of STZ in the STZ group decreased the expression of S100B (Fig. 7a), while *Juglans regia L.* leaf extract treatment in the Preventive and Curative group increased the degree of positive staining for S100B (Fig. 7b) compared to the STZ group. Quantitative analysis assessed by densitometry method using ImageJ software. The histograms of the quantitative analysis of caspase-3, COX-2, and iNOS, and S100B positive staining in the experimental groups are shown in Figs. 4c, 5c, 6c, and 7c respectively.

## Discussion

The hypoglycemic effects of *Juglans regia L.* leaf extract in this study, have been previously proven by the experimental [15–18] and human clinical trial studies [7, 19]. In this regard, the activity was attributed to the antioxidant capacity of the polyphenols present in walnut leaves [31], its effects on glucose-uptake due to inhibition of protein tyrosine phosphatase 1B [20], and its effects on beta cells regeneration and its anti-inflammatory properties [32]. Diabetic neuropathy is the most common complication of diabetes mellitus. On the other hand, free radical induced oxidative stress has been implicated to play an important role in the pathogenesis of diabetic neuropathy [2]. Meanwhile, studies have shown that diabetic neuropathy is a progressive process, despite adequate control of blood sugar, which might be due to accumulation of reactive oxygen species in neural tissue [4]. In diabetic rats, we observed a significant increase in lipid peroxidation, a significant reduction of catalyses and superoxide dismutase activity, and a significant reduction of glutathione content in the sciatic nerve. At the end of the study, treatment with *Juglans regia L.* leaf extract in Preventive and Curative group ameliorated lipid peroxidation and improved antioxidant status in the sciatic nerve of diabetic rats, while the differences between Preventive and Curative were not significant. In addition to hypoglycemic effects, the results indicate that the use of this extract has antioxidative effect even after the onset of neuropathy, considering that oxidative stress after onset of diabetic neuropathy is a progressive process despite adequate control of blood sugar [4]. Free radicals reactive oxygen species (ROS) and reactive nitrogen species (RNS) have been implicated as a potential contributor to the pathogenesis of neurodegenerative diseases such as diabetic neuropathy [33, 34], which seems appropriate for therapeutic interventions such as the use of free radical scavengers. *Juglans regia L.* leaves contain a large amount of phenolic compounds, well-known free radicals scavengers. Phenolic acids, naphthoquinones, and flavonoids are the main phenolic compounds in fresh *Juglans regia L.* leaves [10, 31, 35, 36]. In this regard, study of antioxidant activity of *Juglans regia L.* leaf extract by the reducing power assay and the scavenging effect on DPPH radicals revealed that walnut leaves cultivars have high antioxidant properties [9]. In vitro study indicated that flavonoids from *Juglans regia L.* leaves could reduce the reactive oxygen species level in RAW264.7 cells [10]. Carvalho et al. documented that *Juglans regia L.* leaf extract significantly protected AAPH-induced oxidative hemolysis of human erythrocytes in a time- and concentration-dependent manner [12]. In another study, the antioxidant potential of ethanolic extract of *Juglans regia L.* leaves was measured and the highest ability to chelate Fe^2+^, high reducing power, high antiradical activity, and relatively low prevention of lipid oxidation documented [37]. Results of an in vivo study demonstrated that administration of walnut leaf extract increased the antioxidant enzymes superoxide dismutase and catalase against CCI4-induced oxidative damage in rat liver [38].

Several lines of evidence obtained in experimental and clinical studies demonstrated that inflammation is one of the major reasons behind various deficits seen in diabetic neuropathy. In this regard, it was well documented that the enhanced inflammatory response was mediated by NF-kB axis activation in peripheral nerve disorders caused by diabetes [39]. The NF-kB activation induces cyclooxygenase-2 overexpression, which is implicated in neuropathic changes [40]. For instance, some studies reported that treatment with a COX-2-selective inhibitor and COX-2 gene inactivation prevents functional and biochemical peripheral nerve deficits in the diabetic animals [41, 42]. On the other hand, upregulation of inflammatory mediators of COX-2, iNOS, and TNF-α reported in the course of diabetic neuropathy, blocking of these mediators lead to a marked reduction in pain intensity [43]. Meanwhile, activation of poly (ADP-ribose) polymerase (PARP), a nuclear enzyme which is activated by strand break in DNA, contributes to upregulation on inducible nitric oxide synthase (iNOS) and to nitrosative stress, leading to peroxynitrite formation [44]. Our immunohistocehmical assessments showed that increased COX-2 and iNOS expression in diabetic rats significantly attenuated after treatment with *Juglans regia L.* leaf extract in Preventive and Curative group, while the differences between Preventive and Curative were not significant. In addition to hypoglycemic effects, the results indicate that the extract has anti-inflammatory effects even after the onset of neuropathy, noting that inflammatory response after onset of diabetic neuropathy is a cardinal and progressive process despite adequate blood glucose control [40]. Hosseinzadeh et al. documented that the aqueous and ethanolic extracts of *Juglans regia L.* leaves have anti-inflammatory effect against xylene-induced ear swelling in mice, which is mediated by membrane-stabilizing effect that reduces capillary permeability and/or release of inflammatory mediators [31]. In the other study, it was shown that *Juglans regia L.* leaf extract exhibited anti-inflammatory activity against carrageenan-induced hind paw edema model in mice, the mechanism of the anti-inflammatory activity were not determined [11].

Apoptosis is a key mechanism of degenerative diseases, which is triggered by some factors such as hyperglycemia toxicity. In vivo and in vitro studies revealed that hyperglycemia affected the cell survival and induced apoptotic changes in dorsal root ganglion neurons and Schwann cells [45, 46]. Our immunohistochemical results showed that administration of STZ considerably increased the expression of caspase-3, which plays a critical role in apoptosis. On the contrary, S100B expression, as a Schwann cell marker, decreased in diabetic peripheral neuropathy. Also, our results showed that these up- and down regulations significantly attenuated after *Juglans regia L.* leaf extract consumption in Preventive and Curative group, while the differences between Preventive and Curative group were not significant. Meanwhile, in addition to hypoglycemic effects, our results indicate that the extract has antiapoptotic effects even after the onset of neuropathy, considering that glial apoptosis after onset of diabetic neuropathy is a progressive process despite adequate control of blood sugar [4]. Javidanpour et al. documented the proliferative effects of *Juglans regia L.* leaf extract on pancreatic ß-cells in STZ-induced diabetic rats [15]. Results of another study demonstrated that walnut leaf extract has a hepatoprotective effect against carbon tetrachloride-induced cell death [38]. On the contrary, some studies demonstrated that walnut leaf extract showed a higher antiproliferative efficiency than green husk and seed extracts against various cancer cell line such as human renal, oral, breast and colon cancer cell lines [12, 47], which is more likely related to its phenolic constituents. Also, it was found that walnut extracts suppressed proliferation and induced apoptosis in a dose- and time dependent manner by modulating expression of apoptosis-related genes namely Bax, caspase-3, Bcl2, and tp53 [48, 49]. Sensitized peripheral nociception is a common symptom associated with diabetic neuropathy. Although the exact mechanism of this reaction is unclear, but different mechanisms may account, including peripheral receptor sensitization, ischemic tissue injury, alternation in dorsal root ganglia cells, and sprouting fibers with ectopic activity [50, 51]. In the present study, we used of the formalin-induced nociceptive test because of the induced pain is continuous and, meanwhile it is more similar to the clinical pains versus other nociceptive tests [52]. Acute chemonociception mediated by transient receptor potential vanilloid 1(TRPV1)-expressing nociceptors and prolonged pain provoked by damage of sensory afferents are two distinct mechanisms through which formalin evokes nocifencive behaviors [53]. We observed a significant reduction in nociception after *Juglans regia L.* leaf extract treatment in Preventive and Curative group, while the differences between Preventive and Curative were not significant. Erdemoglu et al. documented that treatment with *Juglans regia L.* leaf extract showed antinociceptive activity against *p*-Benzoquinone-induced abdominal contraction test in mice [11].Also, antinociceptive activity of *Juglans regia L.* leaf extract is further supported by the study of Hosseinzadeh et al. [31], where in central and peripheral antinociceptive effects were documented. These effects may be mediated through non-opioid receptors or inhibition of cyclooxygenase enzyme [31].

## Conclusion

The main findings of the current study showed that administration of *Juglans regia L.* leaf extract attenuates criteria of neuropathy in STZ-induced diabetic rats, in addition to hypoglycemic effects. More importantly, results of this study showed that these beneficial effects are even after the onset of peripheral diabetic neuropathy. In other words, consumption of *Juglans regia L.* leaf extract has preventive and curative effects against STZ-diabetic neuropathy which might be due to its antioxidant, anti-inflammatory, and antiapoptotic properties.

## Abbreviations

AAPH: 2,2′-azo(2-asmidinopropane) dihydrochloride
Bax: BCL2 Associated X Protein
Bcl2: B-cell lymphoma 2
CAT: catalase
COX-2: cyclooxygenase-2
DPN: diabetic peripheral neuropathy
DPPH: α-diphenyl-β-picrylhydrazyl
EGE: advanced glycation end-product
FBG: fast blood glucose
FBS: fast blood sugar
GC-MS: Gas chromatography-mass spectrometry
*GRL*: *Juglans regia L.*
GSH: Glutathione
H&E: hematoxylin and eosin
HbA1c: Hemoglobin A1c
iNOS: inducible nitric oxide synthase
LFB: luxol fast blue
MDA: malondialdehyde
NF-kB: Nuclear factor-kappa-B
PARP: poly (ADP-ribose) polymerase
PBS: Phosphate buffered saline
PTP1B: protein tyrosine phosphatase 1B
RNS: reactive nitrogen species
ROS: reactive oxygen species
S100B: S100 calcium-binding protein B
SOD: superoxide dismutase
STZ: streptozotocin
TNF-α: tumor necrosis factor alpha
tp53: tumor protein p53
*V*/V: volume/volume

## References

[1] Said G (2007). Diabetic neuropathy--a review. Nat Clin Pract Neurol.

[2] van Dam PS. Oxidative stress and diabetic neuropathy: pathophysiological mechanisms and treatment perspectives. Diabetes Metab. 2002;18:176–84. 10.1002/dmrr.287 12112935

[3] Zychowska M, Rojewska E, Przewlocka B, Mika J (2013). Mechanisms and pharmacology of diabetic neuropathy - experimental and clinical studies. Pharmacol Rep.

[4] Oyenihi AB, Ayeleso AO, Mukwevho E, Masola B (2015). Antioxidant strategies in the management of diabetic neuropathy. Biomed Res Int.

[5] Haji Sharifi A. Walnut tree. In secrets of herbal medicine, third ed., Noskh-e-Shafa, Hafez Novin Press, Tehran, Iran, 2003.

[6] Mirheidar H. Walnut tree. Ganjinaeh Asrar Herbal Medicine. Vol. 1., Vahid Press, Tehran, Iran, 2007.

[7] Hosseini S, Jamshidi L, Mehrzadi S, Mohammad K, Najmizadeh AR, Alimoradi H, Huseini HF (2014). Effects of Juglans regia L. leaf extract on hyperglycemia and lipid profiles in type two diabetic patients: a randomized double-blind, placebo-controlled clinical trial. J Ethnopharmacol.

[8] Almeida IF, Fernandes E, Lima JL, Costa PC, Bahia MF (2008). Walnut (Juglans regia) leaf extracts are strong scavengers of pro-oxidant reactive species. Food Chem.

[9] Pereira JA, Oliveira I, Sousa A, Valentão P, Andrade PB, Ferreira IC, Ferreres F, Bento A, Seabra R, Estevinho L (2007). Walnut (Juglans regia L.) leaves: phenolic compounds, antibacterial activity and antioxidant potential of different cultivars. Food Chem Toxicol.

[10] Zhao MH, Jiang ZT, Liu T, Li R (2014). Flavonoids in Juglans regia L leaves and evaluation of in vitro antioxidant activity via intracellular and chemical methods. ScientificWorldJournal.

[11] Erdemoglu N, Küpeli E, Yeşilada E (2003). Anti-inflammatory and antinociceptive activity assessment of plants used as remedy in Turkish folk medicine. J Ethnopharmacol.

[12] Carvalho M, Ferreira PJ, Mendes VS, Silva R, Pereira JA, Jerónimo C, Silva BM (2010). Human cancer cell antiproliferative and antioxidant activities of Juglans regia L. Food Chemical Toxicol.

[13] Rather MA, Dar BA, Dar MY, Wani BA, Shah WA, Bhat BA, Ganai BA, Bhat KA, Anand R, Qurishi MA (2012). Chemical composition, antioxidant and antibacterial activities of the leaf essential oil of Juglans regia L. and its constituents. Phytomedicine.

[14] Noumi E, Snoussi M, Hajlaoui H, Valentin E, Bakhrouf A (2010). Antifungal properties of Salvadora persica and Juglans regia L. extracts against oral Candida strains. Eur J Clin Microbiol Infect Dis.

[15] Javidanpour S, Fatemi Tabtabaei SR, Siahpoosh A, Morovati H, Shahriari A (2012). Comparison of the effects of fresh leaf and peel extracts of walnut (Juglans regia L.) on blood glucose and β-cells of streptozotocin-induced diabetic rats. Vet Res forum.

[16] Jelodar G, Mohsen M, Shahram S (2007). Effect of walnut leaf, coriander and pomegranate on blood glucose and histopathology of pancreas of alloxan induced diabetic rats. Afr J Tradit Complement Altern Med.

[17] Asgary S, Parkhideh S, Solhpour A, Madani H, Mahzouni P, Rahimi P (2008). Effect of ethanolic extract of Juglans regia L. on blood sugar in diabetes-induced rats. J Med Food.

[18] Mohammadi J, Delaviz H, Malekzadeh JM, Roozbehi A (2012). The effect of hydro alcoholic extract of Juglans regia leaves in streptozotocin-nicotinamide induced diabetic rats. Pak J Pharm Sci.

[19] Hosseini S, Huseini HF, Larijani B, Mohammad K, Najmizadeh A, Nourijelyani K, Jamshidi L (2014). The hypoglycemic effect of Juglans regia leaves aqueous extract in diabetic patients: A first human trial. Daru.

[20] Pitschmann A, Zehl M, Atanasov AG, Dirsch VM, Heiss E, Glasl S (2014). Walnut leaf extract inhibits PTP1B and enhances glucose-uptake in vitro. J Ethnopharmacol.

[21] Vandendool H, Kratz PD (1963). A generalization of the retention index system including linear temperature programmed gas-liquid partition chromatography. J Chromatogr.

[22] Morrow TJ. Animal models of painful diabetic neuropathy: the STZ rat model. Curr Protoc Neurosci. 2004;Chapter 9,Unit 9.18. 10.1002/0471142301.ns0918s29 18428614

[23] Sharma SS, Kumar A, Kaundal RK (2008). Protective effects of 4-amino1,8-napthalimide, a poly (ADP-ribose) polymerase inhibitor in experimental diabetic neuropathy. Life Sci.

[24] Erbaş O, Oltulu F, Yılmaz M, Yavaşoğlu A, Taşkıran D (2016). Neuroprotective effects of chronic administration of levetiracetam in a rat model of diabetic neuropathy. Diabetes Res Clin Pract.

[25] Kumar N, Laferriere A, Yu JS, Leavitt A, Coderre TJ (2010). Evidence that pregabalin reduces neuropathic pain by inhibiting the spinal release of glutamate. J Neurochem.

[26] Mihara S, Uchiyama M (1978). Determination of malonaldehyde precursor in tissues by thiobarbituric acid test. Anal Biochem.

[27] Akerboom TP, Sies H (1981). Assay of glutathione, glutathione disulfide, and glutathione mixed disulfides in biological samples. Methods Enzymol.

[28] Aebi H (1984). Catalase in vitro. Methods Enzymol.

[29] Kuthan H, Haussmann HJ, Werringloer JA (1986). Spectrophotometric assay for superoxide dismutase activities in crude tissue fractions. Biochem J.

[30] Kato N, Matsumoto M, Kogawa M, Atkins GJ, Findlay DM, Fujikawa T, Oda H, Ogata M (2013). Critical role of p38 MAPK for regeneration of the sciatic nerve following crush injury *in vivo*. J Neuroinflammation.

[31] Amaral JS, Seabra RM, Andrade PB, Valentao P, Pereira JA, Ferreres F (2004). Phenolic profile in the quality control of walnut (Juglans regia L.) leaves. Food Chem.

[32] Hosseinzadeh H, Zarei H, Taghiabadi E (2011). Antinociceptive, anti-inflammatory and acute toxicity effects of juglans regia L. Leaves in mice. Iranian Red Crescent Med J.

[33] Pacher P, Obrosova IG, Mabley JG, Szabó C (2005). Role of nitrosative stress and peroxynitrite in the pathogenesis of diabetic complications. Emerging new therapeutical strategies. Curr Med Chem.

[34] Vincent AM, Russell JW, Low P, Feldman EL (2004). Oxidative stress in the pathogenesis of diabetic neuropathy. Endocr Rev.

[35] Solar A, Colarič M, Usenik V, Stampar F (2006). Seasonal variations of selected flavonoids, phenolic acids and quinones in annual shoots of common walnut (Juglans regia L.). Plant Sci.

[36] Cosmulescu S, Trandafir I, Nour V (2014). Seasonal variation of the main individual phenolics and juglone in walnut (Juglans regia) leaves. Pharm Biol.

[37] Gawlik-Dziki U, Durak A, Pecio L, Kowalska I (2014). Nutraceutical potential of tinctures from fruits, green husks, and leaves of Juglans regia L. ScientificWorldJournal.

[38] Eidi A, Moghadam JZ, Mortazavi P, Rezazadeh S, Olamafar S (2013). Hepatoprotective effects of Juglans regia extract against CCl4-induced oxidative damage in rats. Pharm Biol.

[39] Wang Y, Schmeichel AM, Iida H, Schmelzer JD, Low PA (2006). Enhanced inflammatory response via activation of NF-kappaB in acute experimental diabetic neuropathy subjected to ischemia-reperfusion injury. J Neurol Sci.

[40] Cameron NF, Cotter MA (2008). Pro-inflammatory mechanisms in diabetic neuropathy: focus on the nuclear factor kappa B pathway. Curr Drug Targets.

[41] Pop-Busui R, Marinescu V, Van Huysen C, Li F, Sullivan K, Greene DA, Larkin D, Stevens MJ (2002). Dissection of metabolic, vascular, and nerve conduction interrelationships in experimental diabetic neuropathy by cyclooxygenase inhibition and acetyl-L-carnitine administration. Diabetes.

[42] Kellogg AP, Pop-Busui R (2005). Peripheral nerve dysfunction in experimental diabetes is mediated by cyclooxygenase-2 and oxidative stress. Antioxid Redox Signal.

[43] Cheng HT, Dauch JR, Oh SS, Hayes JM, Hong Y, Feldman EL (2010). p38 mediates mechanical allodynia in a mouse model of type 2 diabetes. Mol Pain.

[44] Ha HC, Hester LD, Snyder SH (2002). Poly(ADP-ribose) polymerase-1 dependence of stress-induced transcription factors and associated gene expression in glia. Proc Nat Acad Sci USA.

[45] Russell JW, Sullivan KA, Windebank AJ, Herrmann DN, Feldman EL (1999). Neurons undergo apoptosis in animal and cell culture models of diabetes. Neurobiol Dis.

[46] Allen DA, Yaqoob MM, Harwood SM (2005). Mechanisms of high glucose-induced apoptosis and its relationship to diabetic complications. J Nutr Biochem.

[47] Salimi M, Majd A, Sepahdar Z, Azadmanesh K, Irian S, Ardestaniyan MH, Hedayati MH, Rastkari N. Cytotoxicity effects of various Juglans regia (walnut) leaf extracts in human cancer cell lines. Pharm Bio.l 2012;50:1416–22. 10.3109/13880209.2012.682118 22906313

[48] Alshatwi AA, Hasan TN, Shafi G, Syed NA, Al-Assaf AH, Alamri MS, Al-Khalifa AS. Validation of the Antiproliferative Effects of Organic Extracts from the Green Husk of Juglans regia L. on PC-3 Human Prostate Cancer Cells by Assessment of Apoptosis-Related Genes. Evid Based Complement Alternat Med. 2012;2012:103026. 10.1155/2012/103026 PMC3291301 22454652

[49] Hasan TN, B LG, Shafi G, Al-Hazzani AA, Alshatwi AA. Anti-proliferative effects of organic extracts from root bark of Juglans Regia L. (RBJR) on MDA-MB-231 human breast cancer cells: role of Bcl-2/Bax, caspases and Tp53. Asian Pac J Cancer Prev 2011;12:525–530. 21545224

[50] Jensen TS, Baron R (2003). Translation of symptoms and signs into mechanisms in neuropathic pain. Pain.

[51] Quattrini C, Tesfaye S (2003). Understanding the impact of painful diabetic neuropathy. Diabetes Metab Res Rev.

[52] Dubuisson D, Dennis SG (1977). The formalin test: a quantitative study of the analgesic effects of morphine, meperidine, and brain stem stimulation in rats and cats. Pain.

[53] Shields SD, Cavanaugh DJ, Lee H, Anderson DJ, Basbaum AI (2010). Pain behavior in the formalin test persists after ablation of the great majority of C-fiber nociceptors. Pain.

